# The Architecture of Parent-of-Origin Effects in Mice

**DOI:** 10.1016/j.cell.2013.11.043

**Published:** 2014-01-16

**Authors:** Richard Mott, Wei Yuan, Pamela Kaisaki, Xiangchao Gan, James Cleak, Andrew Edwards, Amelie Baud, Jonathan Flint

**Affiliations:** 1 Wellcome Trust Centre for Human Genetics, Roosevelt Drive, Oxford OX3 7BN, UK

## Abstract

The number of imprinted genes in the mammalian genome is predicted to be small, yet we show here, in a survey of 97 traits measured in outbred mice, that most phenotypes display parent-of-origin effects that are partially confounded with family structure. To address this contradiction, using reciprocal F1 crosses, we investigated the effects of knocking out two nonimprinted candidate genes, *Man1a2* and *H2-ab1*, that reside at nonimprinted loci but that show parent-of-origin effects. We show that expression of multiple genes becomes dysregulated in a sex-, tissue-, and parent-of-origin-dependent manner. We provide evidence that nonimprinted genes can generate parent-of-origin effects by interaction with imprinted loci and deduce that the importance of the number of imprinted genes is secondary to their interactions. We propose that this gene network effect may account for some of the missing heritability seen when comparing sibling-based to population-based studies of the phenotypic effects of genetic variants.

## Highlights

• Heritability of murine complex traits has a significant parent-of-origin effect
• Many mouse quantitative trait loci show parent-of-origin effects
• Gene knockouts induce parent-of-origin-like expression changes in reciprocal crosses

## Graphical Abstract

**Figure:**
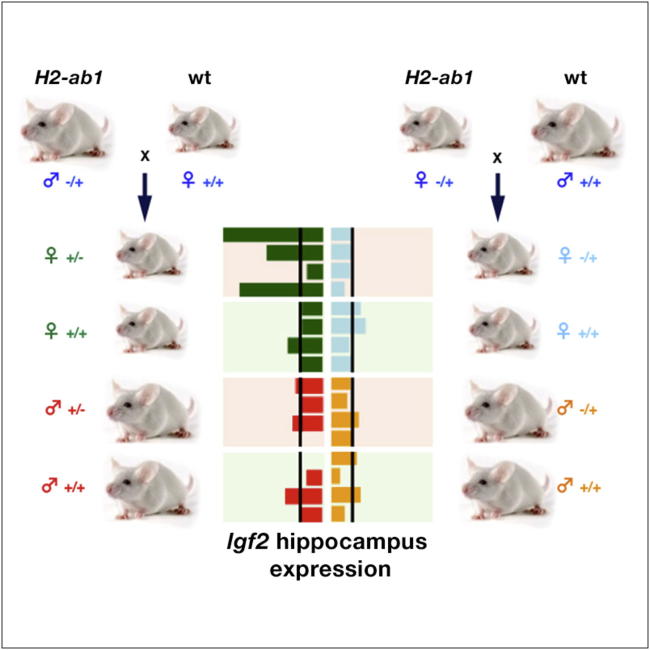

## Teaser

A surprisingly large proportion of traits exhibiting inheritance patterns based on parent of origin indicates a network of interactions between imprinted and nonimprinted genes. The results suggest that these interactions may account for some of the missing heritability seen when comparing sibling-based to population-based studies of the phenotypic effects of genetic variants.

## Introduction

Parent-of-origin effects, in which the phenotypic effect of an allele depends on whether it was inherited from the mother or father, have well-established roles in animal growth (Wolf et al., 2008) and behavior (Garfield et al., 2011). It is less clear whether and how they affect the heritability and genetic architecture of complex traits. Understanding this impact requires a large population of phenotyped individuals of varying degrees of relatedness, whose genotypes are phased with respect to their parent of origin. In one human population in which this has been possible (Iceland), it has emerged as a potentially important contributor to human genetic disease (Kong et al., 2009). Analysis of an advanced intercross in mice has shown that body weight is controlled in a parent-of-origin-specific manner throughout life and that about half of the variance at imprinted quantitative trait loci (iQTLs) is attributable to these effects (Wolf et al., 2008). Similarly, large pedigrees of farm animals have been used to demonstrate significant maternal and paternal influences on growth (e.g., Neugebauer et al., 2010), although in the absence of genotype data.

In this study, in order to estimate the effect of parent of origin on phenotype, we reanalyze data from a mouse heterogeneous stock (HS) previously phenotyped for 97 traits. HS mice are descended from eight inbred progenitor strains (A/J, AKR/J, BALB/cJ, C3H/HeJ, C57BL/6J, CBA/J, DBA/2J, and LP/J) and maintained for over 50 generations (Valdar et al., 2006) so that each HS chromosome is a fine-grained mosaic of the founder haplotypes. QTL mapping in the HS involves the estimation of haplotype trait values (Mott et al., 2000), which lends itself to the analysis of parent of origin: a haplotype has a parent-of-origin effect on a phenotype if its trait value depends on whether it was inherited maternally or paternally, which can be determined where we know the genotypes of the parents of the final mapping generation. Using this population, we estimate the contribution of parent of origin to the heritability of a wide range of complex traits, and identify QTLs with parent-of-origin effects. We then use gene knockouts (KOs) to dissect two QTLs for body weight and CD4^+^ T cells, which do not contain known imprinted genes, to investigate how these effects arise.

## Results

### Effects of Parent of Origin on Heritability

We first show that parent of origin makes a significant contribution to the heritability of most complex traits but is confounded with family structure. We considered 97 traits we had previously analyzed in the HS (Valdar et al., 2006, Solberg et al., 2006). Here, we define a parent-of-origin effect as a difference in the phenotypic effect due to an HS founder haplotype depending on whether it was transmitted via the mother or father in the previous generation. This definition is broader than classical imprinting because it includes phenomena such as polar overdominance (callipyge) (Georges et al., 2003). We used 1,389 of the HS mice for which genotypes at 10,168 SNPs were available for both parents in the immediately preceding generation (212 parents were genotyped in total). The traits and numbers of mice phenotyped for each trait are listed in Table S1, which is available online.

Our analysis is based on the fact that, if two individuals (not necessarily siblings) share an allele, then either it was inherited from parents of the same sex or from parents of the opposite sex. Parent-of-origin effects can then be evaluated by comparing the heritabilities associated with these modes of inheritance. For each mouse and at each locus and for each pair of HS founder haplotypes (s,t), we computed the probability that the animal inherited haplotype *s* maternally and haplotype *t* paternally. We used these phased probabilities to partition the kinship between each pair of HS animals according to parent of origin. Kinship is defined here as the genome-wide average number of shared founder haplotypes. We write + to symbolize coinheritance of an allele from parents of the same sex (i.e., common parent of origin), − for coinheritance from parents of the opposite sex, and ± for coinheritance regardless of parental sex.

**Figure 1:**
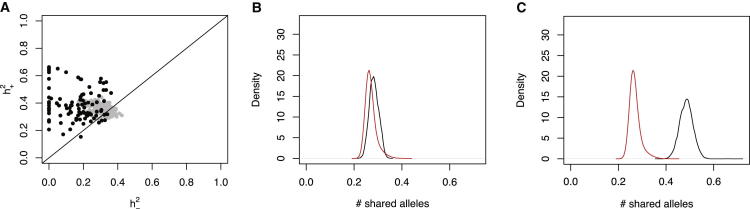
Heritability of Parent-of-Origin Effects (A) Heritability estimates for 97 traits measured in HS mice. Each black dot represents one trait. y axis, the heritability $h_{+}^{2}$ attributed to allele sharing from parents of the same sex; x axis, heritability $h_{-}^{2}$ from parents of the opposite sex. Gray dots are corresponding estimates from simulations of nonimprinted complex traits. The diagonal is the line of equality between the heritabilities. (B and C) Distribution of the parent-of-origin components of kinship between HS mice for siblings (black) and nonsiblings (red). (B) The distribution of the elements of the opposite parent-of-origin kinship matrix $K_{-}$ is shown. (C) Corresponding distribution for the parent-of-origin kinship matrix $K_{+}$ is shown. See also Tables S1 and S2.

The relationships between all HS mice are summarized by the kinship matrix $K_{\pm }$. The kinship of two individuals numbered i,j is the ${\left(i,j\right)}^{\prime }\text{th}$ element of this matrix. In order to investigate parent-of-origin effects, each element of the matrix is partitioned into two components representing the genome-wide average number of haplotypes inherited from parents of the same sex ($K_{+}$), or from parents of opposing sexes ($K_{-}$), so $K_{\pm }=K_{+}+K_{-}$ (see Extended Experimental Procedures). By applying a mixed model commonly used to estimate the heritability of complex traits (Visscher et al., 2006, Yang et al., 2011), we estimated the fractions of phenotypic variation, $h_{+}^{2},\;h_{-}^{2}$ attributable to each component of inheritance (Figure 1A; Table S1). If parent of origin makes no difference, then $h_{+}^{2}=h_{-}^{2}$_,_ and these would each be half the orthodox heritability ignoring parent of origin.

In 91 out of 97 (93%) of the traits, we found $h_{+}^{2}>h_{-}^{2}$ (p value <10^−22^ under the null hypothesis that $h_{+}^{2}=h_{-}^{2}$, one-sided binomial test). Their medians are 0.362 and 0.183, respectively (median ratio $\left(h_{+}^{2}/h_{-}^{2}\right)=2.04$). The median SEs of these estimates are 0.058 and 0.078, respectively. We also estimated heritability from simulated complex traits (each trait generated from seven SNPs selected at random) using the same genotypes and kinship matrices, but without any parent-of-origin effects, and found the median ratio $\left(h_{+}^{2}/h_{-}^{2}\right)=1.08$, as expected (Figure 1A).

We then repeated the analysis using only SNPs within 3 Mb of known imprinted genes (7.5% of all SNPs). Because, in the HS, linkage disequilibrium $R^{2}$ decays to 0.5 within 2 Mb (Valdar et al., 2006), these SNPs capture the genetic signal from imprinted regions. The medians of $h_{+}^{2},\;h_{-}^{2}$ drop to 0.247 and 0.069, respectively (ratio 3.58), apparently suggesting that imprinted regions contribute disproportionately more to $h_{+}^{2}$. However, we observed almost identical results using a circular permutation (Cabrera et al., 2012) for the locations of imprinted genes (medians of $h_{+}^{2},\;h_{-}^{2}$ become 0.248 and 0.084, respectively; ratio 2.95). Randomly chosen loci perform similarly to imprinted loci because both tag the family relationships between HS animals to a similar extent, and hence, the kinship estimated from either subset of the genome approximates the genome-wide kinship equally well. We conclude that, in general, there is no evidence that genetic variation at known imprinted loci explains more parent-of-origin heritability than that expected by chance. We therefore sought other explanations for the observed excess of $h_{+}^{2}$ over $h_{-}^{2}$.

### Parent of Origin Is Confounded with Family Structure

It is well known that siblings share more alleles than nonsiblings. However, it is less appreciated that this excess of shared alleles derives from common parents and so is confounded with parent-of-origin and with parental effects (Hager et al., 2008, Spencer, 2009, Whittaker et al., 2003). The effect is clearly seen when we classify pairs of HS individuals according to whether or not they are siblings (Figures 1B and 1C). The distribution of shared alleles inherited from parents of the opposite sex (i.e., the elements of the matrix $K_{-}$) is nearly identical in siblings and nonsiblings (Figure 1B), whereas parent-of-origin allele sharing $K_{+}$ is much greater between siblings (Figure 1C), and accounts for almost all of the additional genetic similarity among siblings compared to nonsiblings.

Here, allele sharing is defined in terms of the ancestral haplotypes of the HS, but this result applies in any population, for diallelic markers in Hardy-Weinberg equilibrium (see Extended Experimental Procedures). At a SNP with allele frequency p, the average excess of parent-of-origin allele sharing between siblings over nonsiblings is 2p(1−p), whereas average allele sharing from parents of the opposite sex is the same regardless of relationship.

Thus, parent-of-origin allele sharing tracks sibship membership, and the excess of $h_{+}^{2}$ over $h_{-}^{2}$ in the HS is an upper bound on the heritability attributable to parent of origin because it will be confounded with shared environment. Maternal effects are a form of shared environment, so are also confounded. However, because the HS phenotypes analyzed were preprocessed to remove the effects of covariates such as cage that are proxies for shared environment, it is likely that genuine parent-of-origin effects still contribute significantly to the heritability of traits in the HS. Consequently, we expect many QTLs genome wide to also show parent-of-origin effects.

### Parent-of-Origin Effects Occur at Many QTLs

We next investigated whether individual QTLs showed parent-of-origin effects. Here, we define an iQTL to mean a QTL exhibiting an additive parent-of-origin effect. We reanalyzed 837 autosomal QTLs for the 97 traits that we had previously mapped in the HS (Valdar et al., 2006) for parent-of-origin effects. We only considered QTLs previously reported to minimize false-positive calls; however, we will have missed any novel iQTL not possessing a detectable ordinary QTL. We restricted attention to additive parent-of-origin effects and excluded polar overdominance because we had greater power to detect the former (see Extended Experimental Procedures).

We found that standard single-locus tests for parent-of-origin effects, when applied to simulated nonimprinted complex traits, produce many false-positive iQTL calls. This error rate increases with the number of QTLs contributing to the simulated trait, even when a mixed model (Kang et al., 2008) is used to control for relatedness. In addition, the genomic location of the peak of association for an iQTL may shift relative to the peak for the corresponding nonimprinted QTL. This invalidates the use of standard statistical tests to compare models at a single location. For these reasons, we developed a simulation-based methodology to call HS QTLs accurately, as described in the Extended Experimental Procedures. We simulated 1,000 typical complex traits using the HS genotypes, each comprising seven nonimprinted QTLs accounting for 5% of the total variance (i.e., the same simulations represented by the gray dots in Figure 1A). The level of QTL complexity we chose matches that observed in the HS mice and is conservative for this analysis because the median effect size in the HS is only 3% (Valdar et al., 2006). We measured the evidence for an iQTL by the statistic ΔlogP. We estimated the null distribution of ΔlogP for nonimprinted complex traits from the simulations and used this to determine the false discovery rate (FDR) of iQTLs in the real data.

We found 138 iQTLs (16%) with an FDR of 20%, and 304 (36%) at FDR 25% (Table S2). Thus, there is a large fraction of QTLs with weak-to-medium evidence for parent-of-origin effects, although there are relatively few iQTLs of large effect—only 11 at FDR <5%. Over half (60%) of the phenotypes have at least one of the 138 iQTLs. These results are consistent with the observed excess of parent-of-origin heritability affecting most phenotypes, with many loci contributing a small amount to the total.

Some iQTLs overlap known imprinted genes. For example, the imprinting control region between *Dlk* and *Dio3a* (chromosome 12 [chr12], 109.3–114.3 Mb) overlaps five QTLs for diverse phenotypes, four of which are iQTLs at FDR <25%. The region chr7 (58.6–62.5 Mb) contains the *Snord116* cluster syntenic to the human Prader-Willi locus, and overlaps four iQTLs for body weight. However, many iQTLs are not associated with known imprinted regions. For example, another four iQTLs for body weight overlap the locus chr3 (97–101 Mb), yet this contains no known imprinted genes. An iQTL for percentage of CD4^+^ T cells occurs within the major histocompatibility complex (MHC) on chromosome 17. Across all phenotypes, there is no significant enrichment of iQTLs at imprinted genes, although body weight does show slight enrichment (8 out of 10 body weight QTLs that overlap imprinted genes are iQTLs, compared to 39 out of 91 elsewhere; Fisher’s exact test, p < 0.042).

### Parent-of-Origin Effects on HS Gene Expression

**Figure S1:**
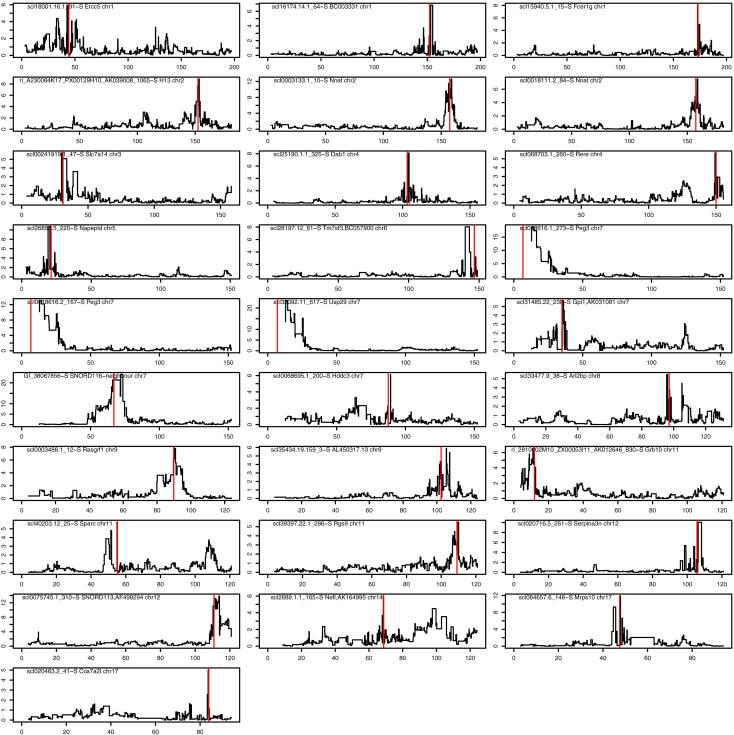
Parent-of-Origin *cis* eQTLs, Related to Table 1 Each of the 28 panels shows a chromosome scan for additive parent of origin effects of an Illumina microarray probe across the chromosome containing the cognate gene. The title of each panel gives the probe name, the gene symbol and the chromosome. The x axis is chromosome position in Mb. The y axis is the negative log10 p value of the test for an additive parent of origin effect in a mixed model (i.e., from a comparison between the fit of an additive nonparent of origin QTL versus an additive parent of origin QTL). The black lines are the chromosome scan logP values. The red vertical line within each panel is the position of the gene.

We next looked for imprinted gene expression effects in the 285 of the 1,389 HS mice for which gene expression microarray data from the hippocampus were available (Huang et al., 2009). Because of the much smaller sample size, and because most gene expression traits have a simpler genetic architecture than complex traits, we limited attention to *cis* eQTLs, and called a *cis* eQTL imprinted if it was within 5 Mb of its gene location (the likely limit distance between a *cis* eQTL and its cognate gene in the HS, based on LD decay in the HS and previous eQTL mapping) and where the test for an additive parent-of-origin effect had logP > 5 in a mixed model. None of the simulated nonimprinted traits reached this level of significance, so we expect fewer than one of the imprinted expression QTLs (ieQTLs) to be a false positive (Table 1; Figure S1).

**Table 1:** *cis* ieQTLs with Parent-of-Origin Effects in the Hippocampus

Probe	Gene	logP	probe.bp	ieQTL.bp
scl18001.16.1_91-S	Ercc5	5.9	chr1: 44,180,973	chr1: 42,492,897–42,492,897
scl16174.14.1_64-S	BC003331	6.8	chr1: 150,362,618	chr1: 151,330,902–151,330,902
scl15940.5.1_15-S	Fcer1g	8.0	chr1: 171,229,809	chr1: 170,841,586–170,841,586
ri_A230084K17_PX00129H10_AK039008_1065-S	H13^a^	8.9	chr2: 152,686,544	chr2: 153,436,538–153,436,538
scl0003133.1_10-S	Nnat^a^	12.6	chr2: 157,561,237	chr2: 158,858,087–158,858,087
scl0018111.2_84-S	Nnat^a^	7.5	chr2: 157,562,196	chr2: 158,858,087–158,858,087
scl00241919.1_47-S	Slc7a14	5.7	chr3: 31,206,501	chr3: 30,147,475–30,147,475
scl25190.1.1_325-S	Dab1	8.1	chr4: 103,712,829	chr4: 104,707,224–104,707,224
scl068703.1_260-S	Rere	5.7	chr4:150621837	chr4: 149,761,825–149,761,825
scl26853.5_220-S	Napepld	10.6	chr5: 21,663,405	chr5: 19,455,955–19,455,955
scl28197.12_81-S	Tm7sf3	8.0	chr6: 146,602,500	chr6: 140,170,891–140,170,891
scl018616.1_273-S	Peg3^a^	18.9	chr7: 6,706,745	chr7: 12,331,090–123,31,090
scl0018616.2_167-S	Peg3^a^	13.0	chr7: 6,707,681	chr7: 12,331,090–12,331,090
scl33092.11_617-S	Usp29^a^	23.5	chr7: 6,967,048	chr7: 12,331,090–12,331,090
scl31485.22_238-S	Gpi1	5.7	chr7: 34,201,350	chr7: 34,914,779–34,914,779
GI_38087856-S	SNORD116 (PWS)^a^	24.0	chr7: 59,676,432	chr7: 64,669,481–64,669,481
scl0068695.1_200-S	Hddc3	6.1	chr7: 80,345,915	chr7: 81,982,400–81,982,400
scl33477.9_38-S	Arl2bp	5.5	chr8: 94,673,938	chr8: 93,133,347–93,133,347
scl0003488.1_12-S	Rasgrf1^a^	7.8	chr9: 89,991,508	chr9: 90,408,780–90,408,780
scl35434.19.159_3-S	Cep63	5.9	chr9: 102,586,724	chr9: 105,639,422–105,639,422
ri_2810002M10_ZX00053I11_AK012646_830-S	Grb10^a^	6.5	chr11: 11,967,505	chr11: 11,116,813–11,116,813
scl40203.12_25-S	Sparc	5.8	chr11: 55,394,523	chr11: 55,151,036–55,151,036
scl39397.22.1_286-S	Rgs9	5.5	chr11: 109,225,377	chr11: 109,419,877–109,419,877
scl020716.5_261-S	Serpina3n	10.0	chr12:104414198	chr12: 105,051,146–105,051,146
scl0075745.1_310-S	SNORD113 (Meg3)^a^	13.8	chr12: 109,652,138	chr12: 112,452,735–112,452,735
scl2689.1.1_165-S	Nefl	6.0	chr14: 68,125,862	chr14: 67,911,078–67,911,078
scl064657.6_146-S	Mrps10	11.9	chr17: 47,378,425	chr17: 47,341,437–47,341,437
scl020463.2_41-S	Cox7a2l	5.0	chr17: 83,502,218	chr17: 83,217,758–83,217,758

Probe is the identification on the Illumina Mouse WG-6 v1 BeadArray. Probe.bp is the mm10 location, gene is the cognate gene or imprinting control region, logP is from the mixed model testing for an additive parent-of-origin effect, and ieQTL.bp the location of the ieQTL peak. The chromosome scans for these data are in Figure S1.

a Known imprinted genes.

We identified 28 ieQTLs representing 26 distinct genes of which 8—*H13*, *Nnat*, *Peg3*, *Usp3*, *Snord116* (Prader-Willi locus), *Rasgrf1*, *Grb10*, and *Snord113* (*Meg3* locus)—are known imprinted genes or in known imprinted control regions. Some of these overlap with the iQTLs we identified, including a probe for a *Snord116* repeat within the Prader-Willi locus (logP = 22) presumably responsible for the parent-of-origin effects on body weight we observed at the locus. Mice with a paternally inherited *Snord116* deletion show severe postnatal growth retardation, whereas maternally inherited deletions are normal (Ding et al., 2008). Similarly, the ieQTLs for *H13* (logP = 8.8) and *Nnat* (logP = 12.6) on chromosome 2 overlap with a single iQTL for body weight. Interestingly, although these ieQTL effects are very significant, the body weight effects are slight. *Grb10* has imprinted behavioral and physiological effects (Garfield et al., 2011) and is close to an ieQTL for body weight on chromosome 11. The other 18 genes with ieQTLs are not known to be imprinted, although 1 (*Ercc5*; logP = 5.94) is potentially imprinted based on expressed sequence tag data (Seoighe et al., 2006). *Ercc5* is an excision repair factor that also promotes DNA breaks and DNA demethylation, allowing the recruitment of the transcription factor CTCF (Le May et al., 2012).

### Causal Genes Underlying iQTLs

**Figure 2:**
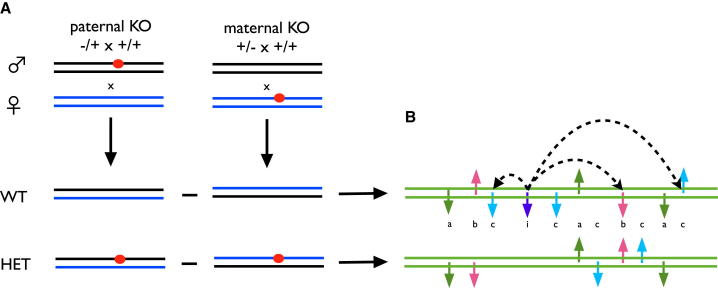
Cartoon of a F1 Reciprocal Cross and Hypothesized Mechanisms Giving Rise to Parent-of-Origin-Dependent Expression Differences Each pair of horizontal lines represents a chromosome pair. (A) The left half of the figure shows crosses between WT and heterozygous KO animals with the mutation present on either paternal (blue) or maternal (black) chromosomes. The KO allele is shown as an orange spot. WT (WT +/+) and heterozygous (HET +/−) offspring are shown below the vertical black arrows. (B) The right half of the figure shows chromosome pairs (green) representing comparisons between mice with a given KO genotype (HET or WT) but with different parent of origin. These are to the right of the corresponding pairs of genomes being compared, linked by the horizontal black arrows. The vertical small arrows on the chromosomes mark genes whose expression depends on the parent of origin of the KO allele. The direction of the arrow indicates expression relative to the maternally derived chromosome. Four phenomena are shown: (a in green) differential expression that is identical in WT and KO comparisons and therefore likely to be driven by parental effects of the KO; (b in pale red) genes that are differentially expressed in both comparisons but in opposite directions, indicating a combination of a parental and parent-of-origin effect; (c in pale blue) differential expression specific to either comparison; and (i in dark blue) a dysregulated imprinted gene whose downstream targets (linked by dashed curved arrows) are differentially expressed.

We next investigated two iQTLs for which there was no underlying imprinted gene and where our ieQTL data did not suggest a candidate. We performed reciprocal F1 crosses between KOs of candidate genes in each of two iQTLs, *Man1a2* (mannosidase α, class 1A, member 2) and *H2-ab1* (histocompatibility 2, class II antigen A, β 1), to confirm function. All laboratory procedures involving mice were conducted under UK Home Office authority after approval by Local Ethical Review at University of Oxford. We distinguish between two types of reciprocal cross: where a homozygote KO is crossed with a wild-type (WT), or where a heterozygote KO is crossed with WT. In the former design, all F1 offspring are heterozygous, and parent-of-origin effects are found by comparing the phenotypes of individuals grouped according to the parent transmitting the KO allele. In the latter (Figure 2A), about half the offspring are WT, with which it is possible to test for purely parental effects, in addition to parent-of-origin effects. For clarity, we denote F1 genotypes as follows: heterozygous F1 mice with a maternally inherited KO allele are denoted by +/−, and heterozygotes with a paternally inherited KO allele by −/+. A WT F1 whose father or mother carried the KO allele without transmitting it is +/+♂ or +/+♀, respectively.

#### Man1a2

**Figure 3:**
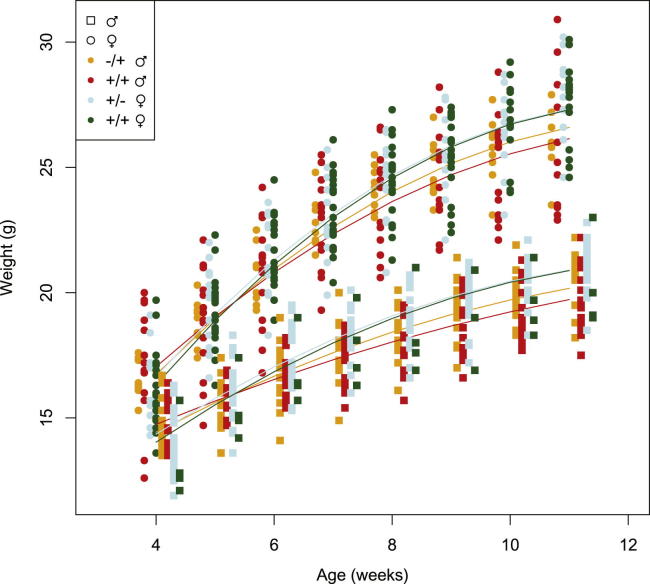
Growth of 108 Offspring from a Reciprocal Cross of *Man1a2* KO Mice Body weight in grams (y axis) is plotted against age in weeks (x axis). For clarity, observations measured at a given age are staggered slightly according to sex (squares versus circles), genotype, and parental genotype: −/+ ♂ (orange), heterozygotes with paternal KO allele; +/+ ♂ (red), WT mice with paternal KO; +/− ♀ (blue), heterozygotes with maternal KO allele; and +/+ ♀ (green), WT mice with maternal KO. The curves show the growth predicted from the linear mixed effects model in Table S3, where weight varies quadratically with age, and depends on sex and on a combined maternal and parent-of-origin effect. Raw weight data are in Table S4. See also Tables S3 and S4.

We first examined the four overlapping iQTLs located around 97–100 Mb on chromosome 3 for body weight measured throughout lifetime. This region contains many genes, but no obvious candidates. We selected the mannosidase *Man1a2*, lying in the middle of the interval, and for which a gene KO B6;129S4-*Man1a2*^*tm1.1Ahe*^/J (Tremblay et al., 2007) was available. Homozygous mutants are nonviable, so we crossed heterozygous KO with WT. Of 108 F1 offspring, 48% were WT. Figure 3 shows the growth curves of the offspring, weighed at ages 4–11 weeks. The ANOVA table for fitting a series of mixed models that dissect the genetic architecture of body weight by sex, genotype, parental genotype, and parent of origin is in Table S3 (body weight data are in Table S4).

Body weight grows approximately quadratically with age, with males heavier than females (p < 10^−170^). The *Man1a2* KO has no direct effect on body weight (p < 0.169) but has two distinct epigenetic effects, which persist throughout life (Wolf et al., 2008). First, and most significantly, there is a purely parental effect, independent of the offspring’s genotype (p < 10^−23^). Second, there is a parent-of-origin effect, where the weight of offspring carrying a KO allele depends on whether it is inherited from the mother or father (p < 0.006).

This combination of effects means there is little difference between KO and WT mice of a given sex whose mother carried the KO allele (blue and green curves in the Figure 3), but KO mice are heavier than WT when the father carries the KO (red and orange curves). We conclude that the paternally inherited *Man1a2* allele has no effect on weight because paternally inherited KO and WT alleles have the same effect.

#### H2-ab1

Theoretical arguments, based on selective abortion of offspring by mothers, suggest that the MHC could be imprinted (Wolf and Hager, 2009). Evidence from studies in rat placenta (Kanbour-Shakir et al., 1993) supports this hypothesis, and in our HS data, immune system traits have a large component of heritability attributed to parent of origin in the HS (Table S1). Moreover, the imprinted minor histocompatibility gene *H13* on chromosome 2 has an HS ieQTL (Table 1). Although we found no immune system iQTL overlapping *H13*, the percentage of CD4^+^ T cells has an iQTL at around 34 Mb on chromosome 17 in the MHC. We therefore investigated whether this could be attributed to genes in the MHC, focusing on *H2-Ab1*. We used the KO B6(C)-*H2*^*bm12*^/KhEgJ, which contains three amino acid substitutions in the β1 exon (Benoist et al., 1983).

**Figure 4:**
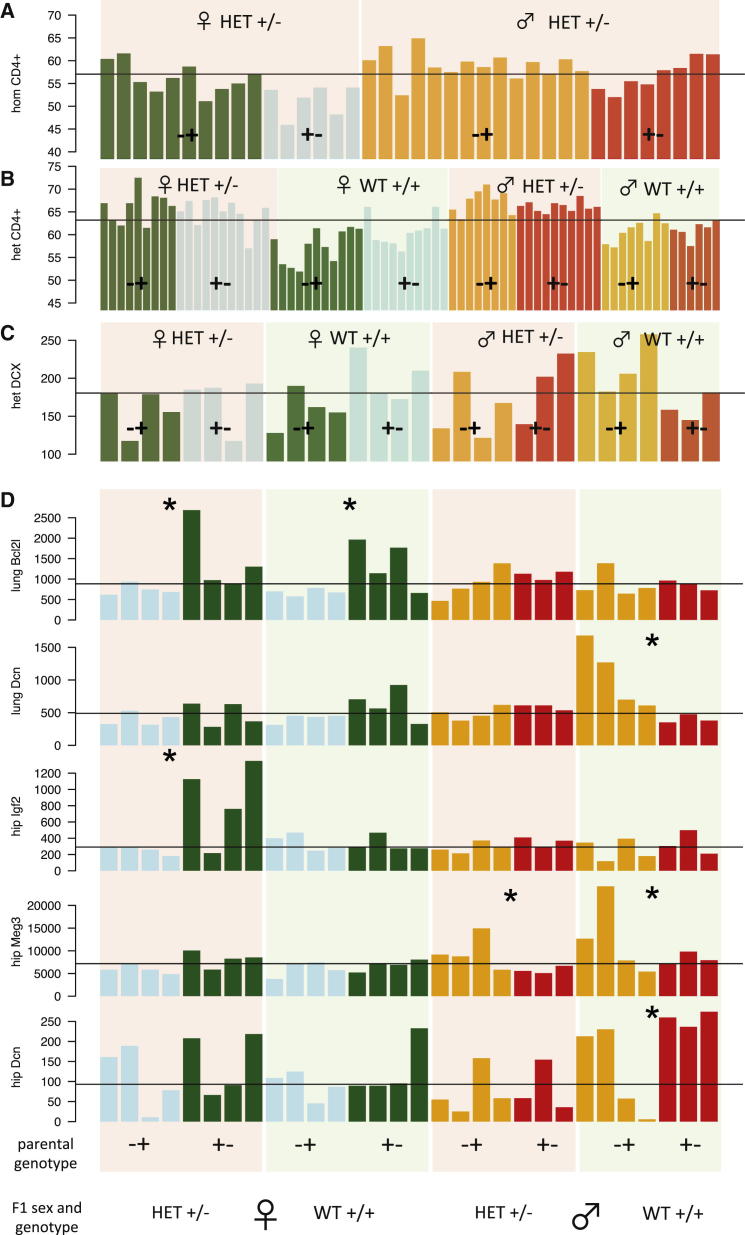
Parent-of-Origin Effects on Phenotypes and Gene Expression in *H2-ab1* Reciprocal Crosses x axis represents individual mice; y axes are phenotype values represented as one bar per animal. Phenotype data are in Table S4. Plots are color coded as in Figure 3. Blue and green bars denote females, and red and orange bars denote males. Blue and orange denote animals where the father carried the *H2-ab1* KO allele, and green and red where the mother carried the KO allele. The pink and green backgrounds denote heterozygous (HET +/−) and WT (WT +/+) F1 animals. The black horizontal lines are the median levels. (A) CD4^+^ T cells in 38 −/− × +/+ *H2-ab1* KO F1 mice (homozygous [hom] cross) are shown. (B) CD4^+^ T cells in 72 +/− × +/+ F1 mice (heterozygous [het] cross) are shown. (C) Neurogenesis, measured by DCX counts in the hippocampi of 30 adult +/− × +/+ F1 mice (heterozygous), is shown. ANOVA tables for these data are in Table S5. Phenotypes are in Table S4. (D) Gene expression across 30 mice for five known imprinted genes (*Bcl2l*, lung; *Dcn*, lung; *Igf2*, hippocampus; *Meg3*, hippocampus; and *Dcn*, hippocampus) is shown. Within each panel, the heights of the bars are the read counts, normalized by library size. Comparisons between sets of three or four mice with different parent of origin but same sex and genotype that DESeq (Anders and Huber, 2010) called as significant at 5% FDR are marked with an asterisk (^∗^). See also Tables S4, S5, S6, and S7.

Homozygous KO animals are viable, so we performed both homozygous and heterozygous reciprocal cross-experiments. In the homozygous experiment, we measured the percentage of CD4^+^ T cells in blood from 38 F1 heterozygous offspring (Figure 4A; Table S5A: data are in Table S4). After accounting for large differences between sexes (p < 0.0009), CD4^+^ T cell levels varied according to parent of origin of the KO allele (p < 0.004). In the heterozygous experiment, 48% of the 73 offspring tested were genotypically WT. We measured CD4^+^ T cells in spleen and blood. In spleen, there was a very significant difference in CD4^+^ T cells between KO and WT F1 mice (Figure 4B; p < 2 × 10^−12^) and a sex difference (p < 0.006). There was a parent-of-origin effect (i.e., an interaction between F1 genotype and parental genotype; p < 0.020), but no parental effect (Figure 4B; Table S5B). There was no parent-of-origin effect in blood.

In both experiments, the parent-origin-effect acted in the same direction, in that heterozygotes that inherited the KO allele from their fathers had lower CD4^+^ T cells than heterozygotes inheriting from the mother. We therefore conclude that *H2-ab1* has a parent-of-origin effect on CD4^+^ T cell levels.

We then reasoned that the *H2-ab1* parent-of-origin effects might also extend to other phenotypes. We investigated neurogenesis, based on the known relationship between T cells and cellular proliferation in the adult hippocampus (Huang et al., 2009). Counts of double cortin (DCX)-stained neurons from 30 adult hippocampi in the heterozygous cross were scored blind in two independent assessments, and the average score was used as a measure of neurogenesis. We found a sex-specific parent-of-origin effect (p < 0.02), but no other significant effects (Figure 4C; Table S5C).

### Parent-of-Origin Effects of *H2-ab1* on Gene Expression

Finally, we looked for parent-of-origin effects on genome-wide gene expression in the *H2-ab1* heterozygous cross. There are two important distinctions between a reciprocal cross in which the only DNA differences segregating are confined to the KO gene—and therefore likely to be casual for all expression differences observed—and one between two inbred strains of mice, where millions of SNPs segregate (Keane et al., 2011) and where there will be many causal variants. In the latter design, imprinting is inferred from allele-specific expression differences tagged by SNPs. In contrast, in our experiment, differential expression of a gene is inferred from a change in its overall expression level, not by allele-specific expression.

We sequenced RNA from 30 F1 mice from the *H2-ab1* heterozygous cross. Both heterozygous and WT animals were sequenced in order to find parental and parent-of-origin effects. We measured expression in the lungs (average 6.2 million reads per sample) and hippocampus (6.6 million reads; the left-brain hemisphere was used to score neurogenesis and the right hemisphere for RNA sequencing [RNA-seq]).

**Figure 5:**
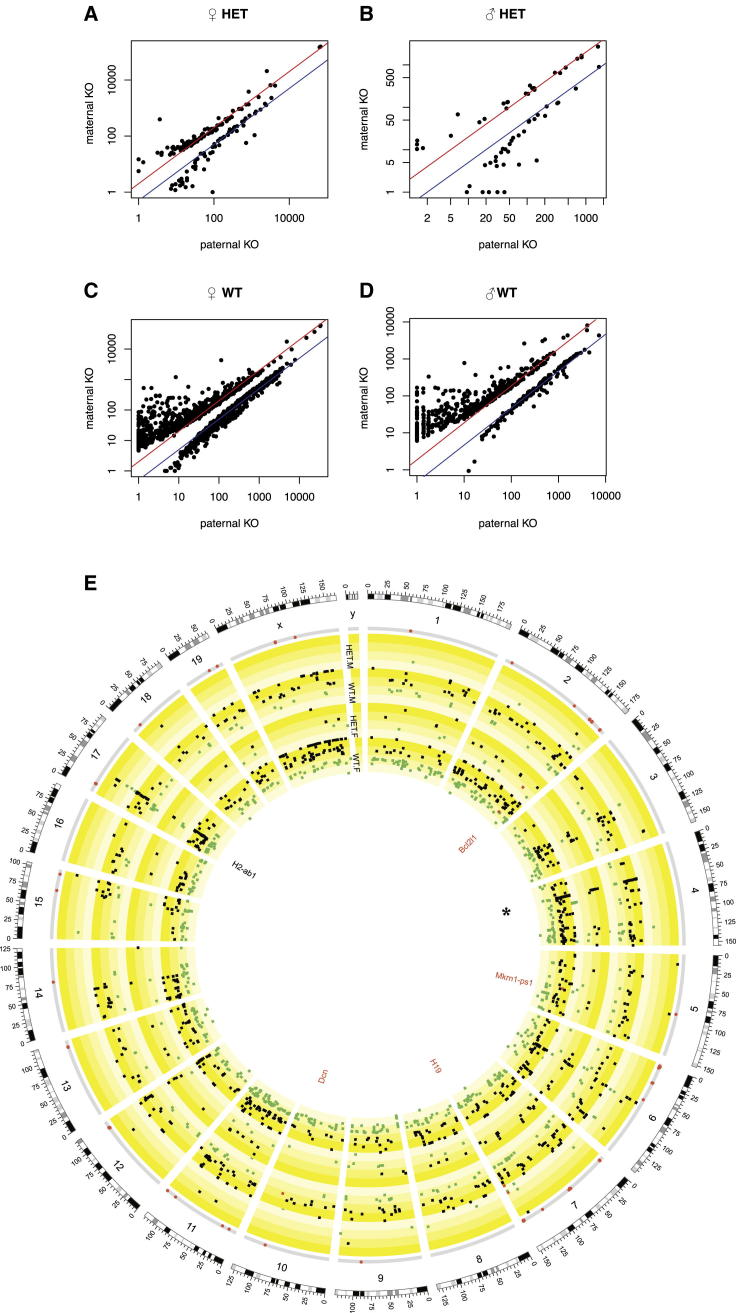
Parent-of-Origin Differential Gene Expression in Lung in Heterozygous +/− × +/+ *H2-ab1* Mice (A–D) Each panel shows mean expression levels of mice where the father carried the KO allele (x axis) versus the mother (y axis). Scales are logarithmic. Each black dot represents one gene. Only genes significant at 5% FDR in each comparison are shown, as determined by DESeq (Anders and Huber, 2010). The red and blue diagonal lines indicate 2-fold up or down expression changes. (A) Females heterozygous for the *H2-ab1* KO. (B) Male heterozygotes. (C) WT females. (D) WT males. See Table S6 for DESeq results and Figure S2 for the corresponding plots in hippocampus. (E) Circos plot shows the genome-wide distribution of differentially expressed genes in (A)–(D) (see Table S6). The outer circle shows the mouse chromosomes. The adjacent thin gray circle marks known imprinted genes by red dots. The next four concentric circles show the positions of differentially expressed genes (at 5% FDR) for the comparisons within heterozygous males (HET.M), WT males (WT.M), heterozygous females (HET.F), and WT females (WT.F). Squares show genes, and radial positions the log_2_ fold change between paternal versus maternal KOs. Gray squares indicate genes with higher expression in mice with a paternal KO, and green squares those with higher expression in mice with a maternal KO. The yellow-shaded subbands show the gradation from maternal to paternal expression. Known imprinted genes that are also differentially expressed are colored red and labeled. The position of *H2-ab1* on chromosome 17 is labeled. The MUP gene cluster on chromosome 4 is marked by an asterisk (^∗^). Overlap between sets of differentially expressed genes is in Table S8. The corresponding circos plot for the *H2-ab1* hippocampus is in Figure S2E. See Tables S6, S7, and S8, and Figure S2.

**Figure S2:**
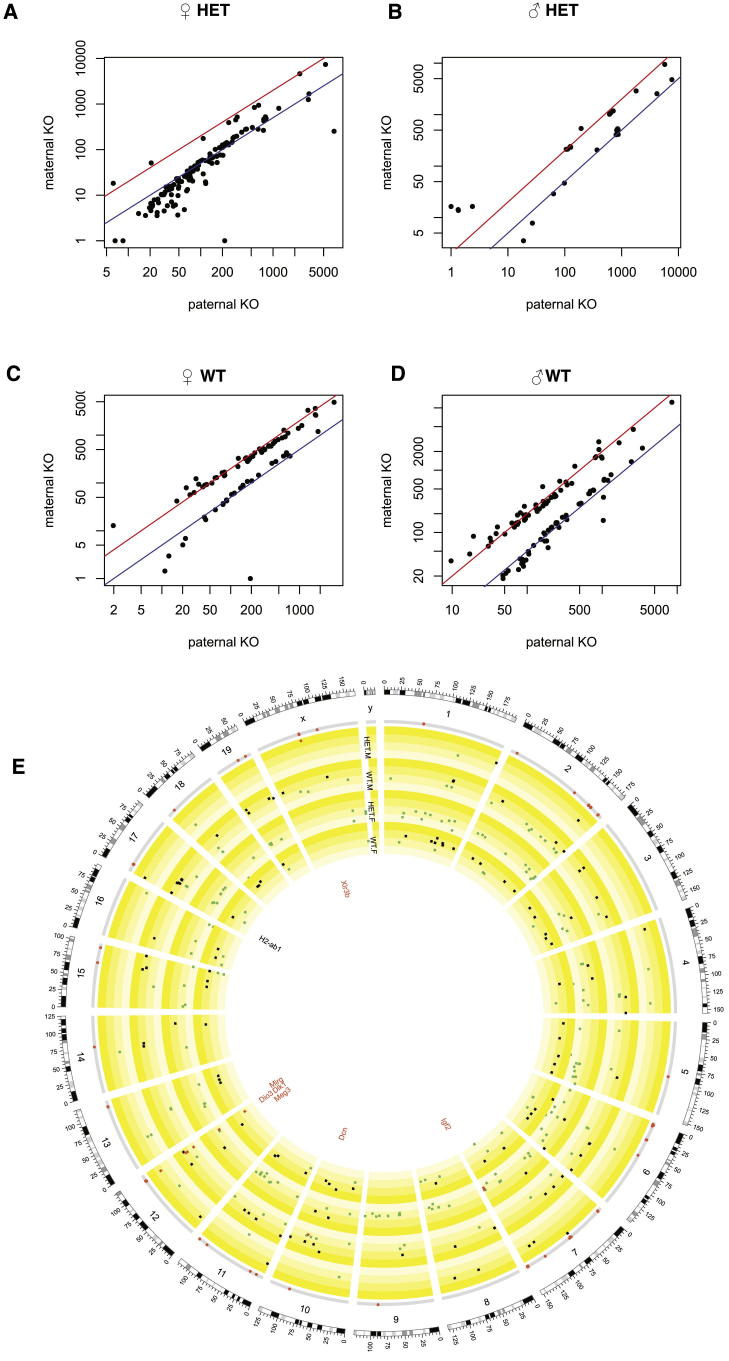
Parent-of-Origin-Dependent Differential Gene Expression for *H2-ab1* Hippocampus, Related to Figure 5 See Figure 5 legend for details. DESeq results and expression counts are in Table S6.

We list in Table S6 those genes that were differentially expressed at FDR < 5%, depending on the parent carrying the KO. We report separate lists classified by sex, tissue, and genotype (i.e., whether the F1 animals were heterozygous or WT). Each list relates to a comparison between two groups of four mice (occasionally three), which except for the sex of the parent carrying the KO are otherwise interchangeable, having the same sex and genotype (see Extended Experimental Procedures). We compare the lung expression levels for mice with paternal KO versus maternal KO in Figures 5A–5D, and for hippocampus in Figures S2A–S2D. The gene expression of individual mice for four differentially expressed known imprinted genes is shown in Figure 4D. We show the genome-wide spatial distribution of differentially expressed genes in the circos plots (Krzywinski et al., 2009) in Figure 5E (lungs) and Figure S2E (hippocampus).

Four general results emerge. First, distinct but overlapping sets of genes are differentially expressed genome-wide depending on sex, tissue, and genotype. In all the comparisons, other genes from within the MHC (and not solely immune system-related genes) are differentially expressed. *H2-ab1* itself is differentially expressed only in the hippocampus of heterozygous females. We conclude that the *H2-ab1* KO exerts a parent-of-origin effect in *cis* and *trans*.

Second, where a gene is differentially expressed, its expression jumps approximately 2-fold up or down, particularly for genes for which expression can be estimated accurately. This is consistent with silencing (or the removal of silencing) of one allele (Figures 5A–5D). Many of these genes are in tandemly repeated genomic clusters: for example, the cluster of major urinary proteins (MUPs) on chromosome 4 is differentially expressed (marked by an asterisk [^∗^] in Figure 5E). MUP genes are normally methylated (Howlett and Reik, 1991), suggesting that the differential expression is caused by loss of methylation.

**Figure 6:**
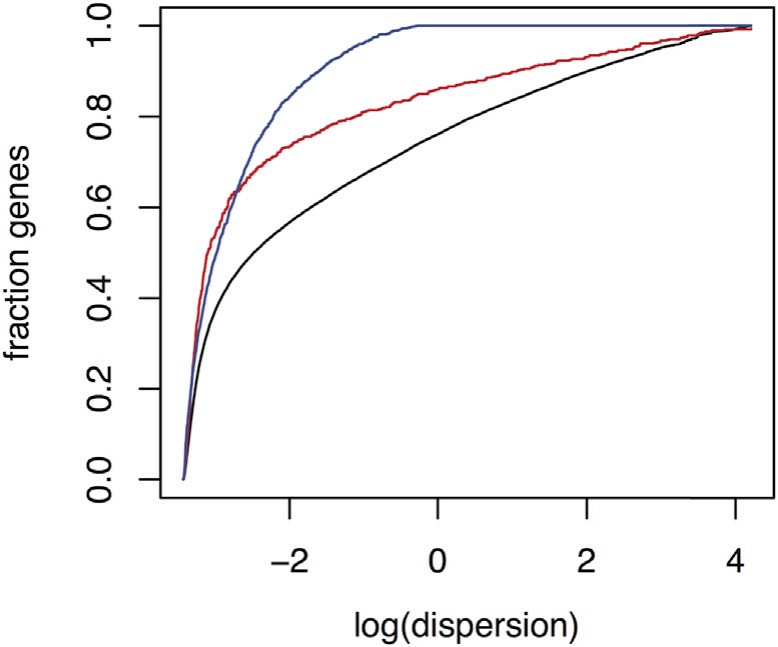
Enrichment of Genes as a Function of Their Gene Expression Dispersion in Lung in the *H2-ab1* Heterozygous Reciprocal Cross The dispersion of a gene is the variance of its expression divided by (1 + mean expression) (see Experimental Procedures). Black indicates cumulative distribution of all expressed genes, blue shows genes significantly differentially expressed in at least one comparison, and red presents cumulative fraction of cancer genes expressed in the tissue. Figure for hippocampus is very similar (data not shown).

Third, differentially expressed genes are under tighter transcriptional control, i.e., variation in expression between mice with the same sex and parental genotype is lower than average (Figure 6). Variation is quantified here by the dispersion, which takes into account that RNA-seq read count data follow a negative binomial distribution approximately. The dispersion s is related to the variance $\sigma^{2}$ and mean μ by $s=\sigma^{2}/\left(\mu +1\right)$, and thus estimates the mean-scaled variance (see Extended Experimental Procedures).

Our data suggest that the dispersion of key genes is under organismal control because cancer-related genes (as defined as the mouse orthologs of the 682 human genes in the human Cancer Gene Census; Futreal et al., 2004) are strongly enriched among those with lowest dispersion (Figure 6). This observation is consistent with the increased variation of DNA methylation seen in cancer (Hansen et al., 2011), which would be expected to lead to greater variation in expression. There is also a statistically significant enrichment of cancer-related genes among the differentially expressed genes in the hippocampus (Fisher’s exact test, p = 2 × 10^−6^). However, this enrichment might also be because we have greater power to detect differential expression in genes with low dispersion.

Finally, some imprinted genes are differentially expressed, suggesting that the *H2-ab1* KO has some form of interaction with imprinted genes, thus accounting for the parent-of-origin effects (Table S7; Figure 4E). For example, in the lung, genes involved in the inflammatory response with a full or partial binding site for *miR-675* are affected, including *Fkbp5* (Kelly et al., 2012), *Cdkn1a*, *Mt2*, *Alox15*, and *Rap1gap*. *miR-675* is generated from the first exon of the imprinted gene *H19* (Keniry et al., 2012), which is differentially expressed in our lung data. *Cdkn1a* affects the expression of many immune-related genes (Fairfax et al., 2012). Similarly, the imprinted gene *Bcl2l1*, which is also differentially expressed in the lung (Table S7), interacts with *Snca* (Nagano et al., 2001), *Atp5o* (Vento et al., 2010), *Fas* (Qin et al., 2002), and *Cycs* (Basañez et al., 2001). Consequently, we conclude that *H2-ab1* generates parent-of-origin effects by dysregulating the expression of known imprinted genes, which then produce a cascade of downstream changes.

## Discussion

We report two observations about parent-of-origin effects in mice: (1) an unexpectedly large component of heritability is attributable to parent of origin, and (2) KOs of two nonimprinted genes under iQTLs produce complex phenotypic and transcriptomic parent-of-origin effects. The interpretation and implications of these findings depend on the way we decompose sources of heritability, which we discuss first.

The HS population contains individuals with differing degrees of relatedness, including both siblings (sharing 50% of their alleles) as well as much more distantly related individuals (sharing less than 5%). We exploit this feature to decompose parental origin effects that remain hidden to designs using unrelated individuals. When we estimate the contribution to heritability attributable to alleles shared by two animals that comes from parents of the same sex (i.e., two mothers or two fathers), we find that it is twice that attributable to shared alleles descended from parents of the opposite sex (one from a mother and one from a father). The former is strongly confounded with family effects because the greater relatedness among siblings derives from identical haplotypes inherited from the same parent. An important implication is that heritability of traits estimated from relatives (in particular twins) will likely exceed those from population-based studies, and this would in part explain why genome-wide SNP-based heritability estimates, and those obtained by summing the effects attributable to detected loci, will underestimate heritability (missing heritability; Manolio et al., 2009).

The second implication is that the contribution of parent-of-origin effects to heritability is unlikely to arise merely from imprinted mono-allelic expression at a locus. Indeed, if mono-allelic expression were the main mechanism responsible for the iQTLs, then many, if not all, would also be detected as ieQTLs. However, whereas we found over 100 iQTLs, we identified only 28 *cis* ieQTLs in the hippocampus. Of course, this might be because we had not assessed expression in the right tissue at the right time, but our F1 cross-results indicate that there are other, more likely, phenomena.

First, the parent-of-origin effect of *Man1a2* on body weight (Figure 2; Table S5) cannot be due to imprinted mono-allelic expression of *Man1a2* because homozygous KOs for this gene are nonviable. The large parental effect suggests that *Man1a2* acts mainly, but not exclusively, through this mechanism. Similarly, parental effects must be responsible for the differential expression observed in WT F1 *H2-ab1* mice (Figures 4D and 5E), which, again, cannot be due to imprinting. In contrast, the ANOVA in Tables S5B and S5C of our *H2-ab1* data on CD4^+^ cells and neurogenesis excludes parental effects.

What mechanisms might explain these data? In both KO and WT animals, differential expression is not continuous but follows approximately either a 2:1 or 1:2 ratio. This is consistent with either an allele being silenced or an inactive allele being restored (and the more extreme values close to the origin in Figures 5A–5D could be interpreted as 1:0 or 0:1 ratios). In the case of the imprinted genes in Figure 4E, differential expression appears to be caused by loss of imprinting resulting in overall increased expression (the example of *Igf2* in hippocampus is particularly striking). These effects are due to maternal or paternal consequences of the *H2-ab1* KO. In contrast, in heterozygous KO mice, the genes that are differentially expressed are largely distinct from the WT set (Tables S6 and S8), so the causal mechanisms cannot be the same.

One explanation is that *H2-ab1* is imprinted and perturbs gene expression independently of any parental effects. A second possibility is that *H2-ab1* is not imprinted, but the presence of the KO allele in the F1 modifies the pre-existing parental effect. That is, there is a statistical interaction between parental genotype and F1 genotype. These two explanations are indistinguishable in our data, but they imply different processes.

If the modifier theory is correct, then one might expect a greater overlap between the sets of differentially expressed genes reflecting a shared mechanism. Overlap analysis is inconclusive: Table S8 counts genes in common between the sets of differentially expressed genes, classified by tissue, sex, and F1 genotype, and whether the direction of differential expression is the same. The fraction of overlapping genes varies but is generally under 10%, and approximately equal numbers are expressed in the same or different directions.

However, the modifier theory is supported by the observation that the sets of differentially expressed genes include known imprinted genes (Table S5) and some of their downstream targets. This observation suggests how nonimprinted genes might produce parent-of-origin effects. We conjecture that where nonimprinted genes interact (in the statistical sense) with imprinted genes, then variation at a nonimprinted locus could acquire imprinted-like effects (Varrault et al., 2006). The idea is outlined in Figure 2B.

One important implication of this mechanism is that sporadic cases of genetic disease might be due to unexpected gene silencing. When an individual inherits only one functional copy of a gene by chance, then the remaining, functional, allele normally rescues the phenotype. But if that functional allele is silenced as a parent-of-origin side effect of a mutation elsewhere in the genome, then both copies become nonfunctional. The individual now carries a null genotype at that locus, which may have phenotypic consequences.

Finally, our results imply that caution must be applied when interpreting gene expression changes in reciprocal crosses between inbred strains. Millions of sequence variants segregate in such experiments: over 16 million (Keane et al., 2011) in the CAST/EiJ × C57BL/6J crosses in Gregg et al. (2010) and DeVeale et al. (2012). It is tacitly assumed that imprinted allele-specific expression is independent of such genetic variation, but we have shown here that when genetic variation inactivates a gene, the variant can have pervasive parent-of-origin effects in *trans* (and over 200 genes are annotated with premature stop codons in CAST/EiJ; Keane et al., 2011). The same may also be true for variants with less-pronounced effects on gene expression, a point that will require additional experimental validation.

## Experimental Procedures

The 1,389 HS mice and their 212 parents used were a subset of those genotyped and phenotyped in Huang et al., 2009, Solberg et al., 2006, and Valdar et al. (2006), for which the genotypes of both parents were available. Statistical analysis of heritability and QTL mapping used the HAPPY R package (Mott et al., 2000) to compute phased haplotype probabilities, from which kinship matrices and iQTLs were computed and analyzed. Heritability was computed using GCTA (Yang et al., 2011) applied to these kinship matrices. Mixed-model QTL analysis used an implementation of the EMMA methodology (Kang et al., 2008) to take account of parent of origin. Phenotype data in F1 crosses were analyzed using the R lme4 package. Read mapping and quantification of RNA-seq data used TopHat (Trapnell et al., 2009). Statistical analysis of RNA-seq data used the DESeq R package (Anders and Huber, 2010).

Heterozygote male and female KO B6;129S4-*Man1a2*^*tm1.1Ahe*^/J *Man1a2*^+/−^ mice (Tremblay et al., 2007) were mated with WT Man1a2 ^+/+^ on a C57BL/6J background, in a reciprocal cross to produce a total of 108 F1 offspring that were genotyped to determine if they were *Man1a2*^*+/−*^, *Man1a2*^*+/−*^, or *Man1a2*^*+/+*^. Animals were weighed every week from age 4 to 11 weeks postbirth. The mice were sacrificed at 11 weeks.

Homozygous and heterozygous KO B6(C)-*H2*^*bm12*^/KhEgJ (Benoist et al., 1983) mice were mated with C57BL/6J WT to produce 38 and 72 F1 offspring, respectively. Animals were sacrificed at 5 weeks, and blood, lung, and hippocampus samples were taken. Blood CD4^+^ T cell levels were measured by FACS. Tissue from lung and the right hippocampus was used for transcriptome measurements, whereas the left hippocampus was used to measure neurogenesis after staining with DCX. Statistical analysis was performed using the R lm() and anova() functions. All data used in this study are available from our website http://mus.well.ox.ac.uk/poe.

Extended Experimental Procedures Data are available from http://mus.well.ox.ac.uk/poe.

**Phased Haplotype Inference via a Hidden Markov Model**

The heterogeneous stock (HS) of mice is descended from eight known inbred strains. For every HS mouse, each chromosome is a mosaic of the founder haplotypes (alleles). The mosaic can be derived from genotypes at a dense set of markers using a hidden Markov model (HMM) in (Mott et al., 2000). We modified the HMM to determine the probability $p_{iL}\left(st\right)$ that at marker L individual i inherits the alleles s,t in phase (i.e., inherits s from the father and t from the mother.

Depending on the genotypes of the parents, at some loci it is possible to determine the phase of the offspring genotype. Thus at the locus *L* in individual *i*, the parental genotypes will convey varying degrees of certainty regarding the phase of the observed unphased genotype g=ab, which can be encoded by the probability $w_{iL}\left(ab\right)$ that the phased genotype is ab rather than ba, so that $w_{iL}\left(ab\right)+w_{iL}\left(ba\right)=1$. If both parents are homozygous but with different genotypes, say aa and bb then the offspring will be perfectly phased with genotype ab so $w_{iL}\left(ab\right)=1$. But if the parents’ genotypes are aa, ab then on average half the offspring will have phased genotype $ab\;\left(w_{iL}\left(ab\right)=1\right)$ and half unphased $aa\;\left(w_{iL}\left(aa\right)=0.5\right)$. If the parents are both heterozygotes then all offspring are unphased. This information can be propagated along the chromosome to compute phased ancestral probabilities as follows:

The forward HMM recurrence (Equation 4 in Mott et al. [2000]) is modified to

$$p_{L-1,i}\left(s,t,a,b\right)=\sum_{\sigma \tau }f_{Li}\left(s,t|\sigma ,\tau ,a,b\right)w_{iL}\left(ab\right)p_{L,i}\left(\sigma ,\tau \right)$$

where $f_{Li}\left(s,t|\sigma ,\tau ,a,b\right)$ is the transition probability that the process jumps from phased state σ,τ at *L*-1 to phased state s,t at *L*, given the phased genotype a,b at *L*. The backward recurrence is defined similarly, from which we can compute the probability $P_{iL}\left(st\right)$ be that at marker interval L, the individual i inherits the alleles s,t in phase (i.e., inherits s from the father and t from the mother.

**Allele Sharing in HS Mice**

We next compute $\pi_{ijL}\left(k\right)$, the probability that k=0,1,2 alleles at locus L are shared between individuals i,j via parents of the same sex. Here allele means one of the ancestral HS haplotypes. Let $\phi_{iL}\left(s\right)=P_{iL}\left(s\cdot \right)=\Sigma_{t}\;P_{iL}\left(st\right)$ be the probability the allele s was inherited from the father, and $\psi_{iL}\left(t\right)=P_{iL}\left(\cdot \;t\right)=\Sigma_{s}\;P_{iL}\left(st\right)$ the probability the allele t was inherited from the mother. Then

$$\pi_{ijL}\left(2\right)=\sum_{st}P_{iL}\left(st\right)P_{jL}\left(st\right)$$

$$\pi_{ijL}\left(1\right)=\sum_{s}\sum_{t\neq t^{'}}P_{iL}\left(st\right)P_{jL}\left(st^{\prime }\right)+\sum_{t}\sum_{s\neq s^{\prime }}P_{iL}\left(st\right)P_{jL}\left(s^{\prime }t\right)$$

The first term above is the probability A of sharing exactly one allele paternally and the second term is the probability B of sharing exactly one allele maternally. The paternal probability A is equal to

$$A=\sum_{st}\;P_{iL}\left(st\right)\left\{P_{jL}\left(s\cdot \right)-P_{jL}\left(st\right)\right\}=\Sigma_{s}\;P_{iL}\left(s\cdot \right)P_{jL}\left(s\cdot \right)-\pi_{ijL}\left(2\right)$$

$$A=\sum_{s}\phi_{iL}\left(s\right)\phi_{jL}\left(s\right)-\pi_{ijL}\left(2\right)$$

Similarly

$$B=\sum_{t}\psi_{iL}\left(t\right)\psi_{jL}\left(t\right)-\pi_{ijL}\left(2\right)$$

so that

$$\pi_{ijL}\left(1\right)=\sum_{s}\phi_{iL}\left(s\right)\phi_{jL}\left(s\right)+\Sigma_{t}\psi_{iL}\left(t\right)\psi_{jL}\left(t\right)-2\pi_{ijL}\left(2\right)$$

Then the expected number of common alleles inherited from a parent of the same sex is $E_{ijL}=0.\pi_{ijL}\left(0\right)+1.\pi_{ijL}\left(1\right)+2.\pi_{ijL}\left(2\right)$, i.e.

$E_{ijL}=\Sigma_{s}\phi_{iL}\left(s\right)\phi_{jL}\left(s\right)+\Sigma_{t}\psi_{iL}\left(t\right)\psi_{jL}\left(t\right)=\phi_{\mathrm{iL}}\cdot \phi_{\mathrm{jL}}+\psi_{\mathrm{iL}}\cdot \psi_{\mathrm{jL}}$, using vector dot-product notation. By a similar argument, the expected number of shared alleles inherited from parents of opposite sex is $F_{ijL}=\phi_{\mathrm{iL}}\cdot \psi_{\mathrm{jL}}+\psi_{\mathrm{iL}}\cdot \phi_{\mathrm{jL}}$.

Consequently, because any common alleles must have been inherited either from parents of the same sex or opposite sex, the expected number of common alleles regardless of parent of origin is

$$K_{ijL}=E_{ijL}+F_{ijL}=\phi_{\mathrm{iL}}\cdot \phi_{\mathrm{jL}}+\psi_{\mathrm{iL}}\cdot \psi_{\mathrm{jL}}+\phi_{\mathrm{iL}}\cdot \psi_{\mathrm{jL}}+\psi_{\mathrm{iL}}\cdot \phi_{\mathrm{jL}}$$

$$K_{ijL}=\left(\phi_{\mathrm{iL}}+\psi_{\mathrm{jL}}\right)\cdot \left(\psi_{\mathrm{iL}}+\phi_{\mathrm{jL}}\right)$$

Write $\Phi_{L}$ for the matrix whose *i* th column is $\phi_{iL}$, and $\Psi_{L}$ for the matrix whose *i* th column is $\psi_{\mathrm{iL}}$. Then we can write the matrices $E_{L},F_{L},K_{L}$ whose *i*, *j* th elements are $E_{ijL},\;F_{ijL},\;K_{ijL}$ as

$$E_{L}=\Phi_{L}^{\prime }\Phi_{L}+\Psi_{L}^{\prime }\Psi_{L}$$

$$F_{L}=\Phi_{L}^{\prime }\Psi_{L}+\Psi_{L}^{\prime }\Phi_{L}$$

$$K_{L}=\left(\Phi_{L}^{\prime }+\Psi_{L}^{\prime }\right)\left(\Psi_{L}+\Phi_{L}\right)$$

Finally by averaging across all *N* loci *L*, and thereby dropping the subscript *L*, and using “+” to denote haplotype sharing from a parent of the same sex, and “-“ haplotype sharing from parents of opposite sexes, we obtain the genome-wide kinship matrices

$$E=K_{+}=\frac{1}{N}\sum_{L}\Phi_{L}^{\prime }\Phi_{L}+\Psi_{L}^{\prime }\Psi_{L}$$

$$F=K_{-}=\frac{1}{N}\sum_{L}\Phi_{L}^{\prime }\Psi_{L}+\Psi_{L}^{\prime }\Phi_{L}$$

$$K_{\pm }=K_{+}+K_{-}=\frac{1}{N}\sum_{L}\left(\Phi_{L}^{\prime }+\Psi_{L}^{\prime }\right)\left(\Psi_{L}+\Phi_{L}\right)$$

Note that, in general the diagonal elements of $K_{+},K_{-},K_{\pm }$ are not unity unless all the probabilities $P_{iL}\left(st\right)$ are either 0 or 1 (i.e., the haplotypes are known with complete certainty). Therefore $K_{+},K_{-},K_{\pm }$ are scaled to make the diagonal elements of $K_{\pm }$ all equal to unity, by the transformation K=DKD where D is the diagonal matrix whose element $D_{ii}=1/\sqrt{K_{\pm ii}}$. The same transformation is applied to $K_{+},K_{-}$ so that $K_{\pm }=K_{+}+K_{-}$

These matrices were computed from the HMM probabilities in R and saved as files.

**Parent-of-Origin Allele Sharing at a SNP in Hardy-Weinberg Equilibrium**

Consider a SNP with allele frequencies p+q=1. For two siblings, the probability that their maternally inherited allele is identical is

$$\pi_{m}=\mathrm{Pr}\left(\text{mother}\;\text{homozygous}\right)+0.5*\mathrm{Pr}\left(\text{mother}\;\text{heterozygous}\right)$$

$$\pi_{m}=\left(p^{2}+q^{2}\right)+0.5*2pq=p^{2}+q^{2}+pq.$$

The probability $\pi_{p}$ the paternally inherited allele is identical is the same as $\pi_{m}$. Hence for two siblings the expected number of identical alleles inherited from a parent of the same sex is $K_{+}^{sib}=2\left(p^{2}+q^{2}+pq\right)$.

For unrelated nonsiblings the parental genotypes are independent. Let $\pi_{m}=\mathrm{Pr}\left(\text{both mothers}\;\text{transmit}\;\text{same}\;\text{allele}\right)$. Then

$\pi_{m}=(p^{2}+p{q)}^{2}+(q^{2}+p{q)}^{2}=p^{2}+q^{2}$, because $p^{2}+pq$ is the probability either mother transmits the allele corresponding to frequency *p*, and the transmissions are independent. The paternal case has the same probability. From this it follows that $K_{+}^{nonsib}=2\left(p^{2}+q^{2}\right)$, and that $k_{+}^{sib}-k_{+}^{nonsib}=2pq$, so $0.5\geq k_{+}^{sib}-k_{+}^{nonsib}\geq 0$, with the maximum at *p* = 0.5.

**Heritability Estimation**

Under the above kinship structure, a phenotype vector y has variance-covariance matrix $\mathrm{var}\left(y\right)=K_{+}\sigma_{g+}^{2}+K_{-}\sigma_{g-}^{2}+I\sigma_{e}^{2},$ where $\sigma_{g+}^{2}$ is the variance attributed to common parent of origin, $\sigma_{g-}^{2}$ is the variance attributed to opposite parent of origin and $\sigma_{e}^{2}$ is the environmental variance. If $\sigma_{g+}^{2}=\sigma_{g-}^{2}=\sigma_{g}^{2}$ then the covariance structure reduces to that of the standard kinship model $\mathrm{var}\left(y\right)=K_{\pm }\sigma_{g}^{2}+I\sigma_{e}^{2}$. Heritabilities were estimated with GCTA (Yang et al., 2011) using the above kinship matrices. Each phenotype was first transformed to residuals by removing those covariates (such as sex and cage) that were significantly associated with the trait.

**iQTL Mapping**

We used the phased probabilities $P_{iL}\left(st\right)$ to test for parent of origin effects at QTLs we had previously detected. In general $P_{iL}\left(st\right)$ and $P_{iL}\left(ts\right)$ are unequal and this difference can be used to test for a parent of origin effect. Under the null hypothesis of no parent of origin effect at locus L, let the additive effect on the phenotype of the QTL haplotype s be $\beta_{s}$, so the combined additive effect of haplotypes s,t is $\beta_{s}+\beta_{t}$.

Under the alternative hypothesis that there is a parent of origin effect, let $\alpha_{s}$ be the effect of a maternally inherited haplotype *s*, and $\gamma_{s}$ the effect of a paternally inherited haplotype s. Noting that $\beta_{s}=\alpha_{s}+\gamma_{s}$ and letting $2R_{iL}\left(st\right)=P_{iL}\left(st\right)+P_{iL}\left(ts\right)$ when s,t are unequal, we compare the fits of the no-parent of origin additive linear regression model

(1) $$E\left(y_{i}\right)=\mu_{i}+\Sigma_{st}2R_{iL}\left(st\right)\left(\beta_{s}+\beta_{t}\right)$$

with the parent of origin model

(2) $$E\left(y_{i}\right)=\mu_{i}+\Sigma_{st}P_{iL}\left(st\right)\left(\alpha_{s}+\gamma_{t}\right)$$

where $y_{i}$ is the phenotype of animal i and $\mu_{i}$ is the expected nongenetic effect of covariates such as sex on animal i.

The no-parent of origin model (1) is nested within the imprinted model (2), and model (2) contains twice the number of haplotype parameters as model (1). In principle the evidence for imprinting can be tested at each locus L using a partial F-test to compare the models. However, we found this is unreliable for two reasons. First, at a typical QTL where we might test 10 or more consecutive locus intervals, the position of the maximum association for model (1) need not coincide with that for model (2), making it difficult to determine if the effect was real as the models would no longer be nested. Second, and more importantly, extensive simulations of complex traits (each trait comprising seven 5% QTLs) showed that the confounding of family structure with parent of origin resulted in many false positive partial F-test test statistics, and therefore making it impossible to call parent of origin effects reliably in this way.

Instead we used the simulations to derive the empirical distribution of a statistic ΔlogP under the null hypothesis that a QTL is caused by a complex trait without parent of origin effects. We fitted a mixed model within every SNP interval inside each QTL (a QTL typically has about ten SNP intervals [Valdar et al., 2006]). The mixed model used the nonparent of origin kinship matrix $K_{\pm }$ to control for family structure. We used the R EMMA package to estimate the variance matrix $V=\mathrm{var}\left(y\right)=K_{\pm }\sigma_{g\pm }^{2}+I\sigma_{e}^{2}$ (Kang et al., 2008). Then we decomposed into its matrix square root: $V=W^{2}$ and premultiplied the linear models (1) (2) by $W^{-1}$ to remove genetic correlations (the standard EMMA methodology). Thus each transformed phenotype resembled a sample from a standard phenotypic distribution, from a population of equally related individuals. This implicitly removed the effects due to variance differences between phenotypes.

The evidence for an iQTL was summarized by the statistic $\Delta \log P=\max_{L}\;\log\;P_{L}^{+}-\max_{L}\;\log\;P_{L}^{\pm }$, where $\log\;P_{L}^{+}$ is the negative log_10_ p value of the imprinted QTL fit at locus *L* (i.e. model (2)) and $\log\;P_{L}^{\pm }$ the logP of the nonimprinted QTL fit at *L* (model (1)), and where *L* runs over each interval across the QTL. The null distribution of ΔlogP for nonimprinted complex traits was estimated from 1,000 simulations of complex traits similar to those above (i.e., 7000 QTLs in total), and transformed under the above mixed model methodology to remove variance differences. From these we derived the empirical distribution of ΔlogP and used this to determine probable iQTL.

We required parent of origin effects to be additive, which reduced the parameter space considerably (there are 64 combinations of 8 founder haplotypes in the most general parent of origin model in which these effects can be nonadditive, and which include polar overdominance. There are only 16 combinations under an additive parent of origin model). We also tested for iQTL using the most general formulation for an iQTL (data not shown). However, comparisons with our simulated QTLs showed that we had a lower false discovery rate (FDR) when the additive iQTLs were called rather than in the more general model.

**ieQTL Analysis**

We used the hippocampus Illumina microarray gene expression data in Huang et al. (2009). Expression QTLs were tested for parent of origin effects in a similar framework to ordinary QTLs, with the following difference: First, because expression traits have far fewer expression QTLs than do complex traits (typically just one *cis* eQTL), and therefore have a simpler genetic architecture, we compared observed ieQTLs to simulations where each trait had a single QTL explaining 5% of the variance. For these simulations we did not observe the same confounding of parent of origin with family effects and therefore did not need to use the statistic ΔlogP. Instead, we scanned the chromosome containing the cognate gene to the microarray probe and compared the fits of mixed models with and without parent of origin effects at each locus. We then reported those probes for which the negative log10 p value of the comparison was greater than 5 and occurred within 5 Mb of the cognate gene (see Figure S1. No simulated traits were observed to exceed this threshold.

**Reciprocal F1 Cross-Analysis**

All mice were housed and procedures carried out at the WTCHG. Mice were maintained in a specific pathogen free facility in individually ventilated cages, and given food and water *ad libitum*. All experiments were carried out in accordance with UK national (Home Office) and institutional guidelines.

**Man1a2**

Exploratory data analysis showed that body weight had an approximately quadratic dependence on age. We analyzed weights from all mice at all time points in a single linear mixed model in order to parameterize the growth curves. Repeated observations on a given animal are correlated, so a random effect for each mouse was included in the model. A series of nested models of increasing complexity was fitted, in order to dissect the effects of sex, parent and genotype on weight. Thus, using the R formula terminology, a model that describes weight w in terms of sex s, parental genotype p and offspring genotype g with a quadratic dependence on age a, with a random effect for each mouse (m), has the R formula

$$w∼\left(a+a^{2}\right)*\left(s*p*g\right)+\left(1|m\right)$$

Mixed models were fitted using the lmer() function in the R lme4 package and differences between nested models in Table S3 were tested using the R anova() function.

**H2-ab1**

CD4^+^ T cells were measured in blood and spleen and Double cortin (DCX) stained neurogenesis were measured in hippocampus using the protocols in (Huang et al., 2010). Tests for parent of origin effects were made using the R lm() and anova() functions.

**F1 Reciprocal Cross-RNA-Seq**

All RNA sequencing was performed at the WTCHG Oxford Genomics Centre.

Hippocampus and lung RNA was extracted from 30 of the H2-ab1 mice from the second experiment (heterozygote x wild-type). Total RNA was isolated from frozen lung using RNeasy kit, and from frozen hippocampus using RNeasy micro kit (QIAGEN, Crawley, UK). RNA concentrations were determined using a NanoDrop spectrophotometer, and lung RNA quality, purity and integrity were assessed using an Agilent 2100 Bioanalyser (Agilent Technologies, Waldbronn, Germany). Hippocampus RNA quality was not routinely assessed on the Bioanalyser because the quantities were too low. Three or (usually) four mice from each combination of sex, F1 genotype and parental genotype were used.

RNA was barcoded, pooled and sequenced on a lane of a Hiseq2000 using 51bp paired end reads. mRNA-seq library construction was as follows: Total RNA quantity and integrity were assessed, using Quant-IT RiboGreen RNA Assay Kit (Invitrogen, Carlsbad, CA, USA) and Agilent Tapestation 2200 R6K. mRNA enrichment was achieved by processing 5μg of total RNA using the Magnetic mRNA Isolation Kit from NEB (S1550S) with minor modifications. Generation of double stranded cDNA and library construction were performed using NEBNext Ultra Directional RNA Library Prep Kit for Illumina® (E7420L) according to manufacturer specifications.

Upon ligation of Illumina Adapters (Multiplexing Sample Preparation Oligonucleotide Kit) each library was size selected with two Ampure Bead bindings (first, 1:0.7x volume and second, the supernatant from the first bind was taken for a 1:1.7x volume clean-up). The following custom primers (25 μM each) were used for the PCR enrichment step:

Multiplex PCR primer 1.0

5′-AATGATACGGCGACCACCGAGATCTACACTCTTTCCCTACACGACGCTCTTCCGATCT-3′

Index primer

5′-CAAGCAGAAGACGGCATACGAGAT[**INDEX**]CAGTGACTGGAGTTCAGACGTGTGCTCTTCCGATCT-3′

Barcoding indices used were the eight base tags developed by (Lamble et al., 2013).

Amplified libraries were analyzed for size distribution using the Agilent Tapestation 2200 D1K. Libraries were quantified by quantitative RT-PCR using Agilent qPCR Library Quantification Kit and a MX3005P instrument (Agilent) and relative volumes were pooled accordingly. Finally, a second quantitative RT-PCR was performed to measure the relative concentration of the pool compared to a previously sequenced mRNA library in order to determine the volume to use for sequencing. Sequencing was performed as 50bp paired end read on a HiSeq2000 according to Illumina specifications.

**F1 Reciprocal Cross-RNA-Seq Analysis**

After standard quality control reads were aligned to the mouse reference genome (mm10) using TopHat (Trapnell et al., 2009) with default parameters. The read counts per Ensembl gene were computed using the R Bioconductor (http://bioconductor.org) packages Genomic Ranges, Rsamtools, GenomicFeatures, rtracklayer, leeBamViews. Count data are in Table S6 and are also available from ArrayExpress (one per tissue, H2-ab1.hip.gene.counts.txt, H2-ab1.lung.gene.counts.txt) where the rows are genes and columns are individual mice. Differential expression determined using DESeq (Anders and Huber, 2010). We found the most sensitive parameter settings for the DESeq function estimateDispersions() were method = ”blind,” sharingMode = ”fit-only.”

## Accession Numbers

The ENA (European Nucleotide Archive) and Array Express accession numbers for the RNA sequence data reported in this paper are ERP004119 and E-MTAB-1998, respectively.

## Supplemental Information

Document S1. Tables S1, S3, S5, S7, and S8

Table S2. QTLs with Evidence of Parent-of-Origin Effects, Related to Figure 1 False discovery rates (FDR) for 837 QTLs based on comparison of the statistic ΔlogP to simulated data. QTLs are sorted by genome location (mm9).

Table S4. Raw Phenotypic Data from Reciprocal Cross-Experiments, Related to Figures 3 and 4 The Excel spreadsheet contains three workbooks. (a) Body weight data for the *Man1a2* cross. mouse: identifier of mouse, sex:sex of mouse, parental.genotype: whether mother or father carried the KO allele, F1.genotype: whether mouse carried the KO allele (HET) or was wild-type (WT), age: age in weeks of mouse at weighing, weight: body weight in g. Relates to Figure 3 (b) CD4^+^ T cells for the *H2-ab1* homozygous cross. (c) CD4^+^ T cells for the *H2-ab1* heterozygous cross. Relates to Figure 4. (d) Double cortin (DCX) counts for the *H2-ab1* heterozygous cross.

Table S6. Differential Gene Expression in Lung and Hippocampus in the *H2-ab1* Reciprocal Cross, Related to Figures 4E, 5, and 6 The Excel spreadsheet contains 12 workbooks. For Worksbooks 1-8, each lists those genes differentially expressed at FDR < 5% in a particular combination of tissue (hip = hippocampus or lung), *H2-ab1* genotype (KO = heterozygous knockout, WT = wild type), sex (M = Male, F = Female) e.g., hip HET.F means the comparison for female mice heterozygous for the *H2-ab1* knockout in the hippocampus, between individuals whose father carries the knockout and those whose mother carried it. Workbooks 9, 11 contain the counts of reads aligned to each Ensembl gene, for lung and hippocampus. Columns are mice, rows are genes. Workbooks 10,12 contain the sample sheets needs to decode the genotype, sex and parental genotypes of the mouse ids in workbooks 9,11.

Document S2. Article plus Supplemental Information
