# Supplementary material for: The Architecture of Parent-of-Origin Effects in Mice
Source: Cell. 2014 Jan 16;156(1-2):332–42. doi: 10.1016/j.cell.2013.11.043 (PMC3898482; doi:10.1016/j.cell.2013.11.043)
Supplement: Document S1. Tables S1, S3, S5, S7, and S8 [file mmc1.pdf]

Table S1. Estimates of Heritability, Related to Figure 1

**Trait**	h_+^2	h_-^2	$\Delta(h_+^2)$	$\Delta(h_-^2)$	N
AUC.IRI.AUC.G	0.33	0.18	0.06	0.08	938
AdrenalMeanWeight	0.31	0.22	0.04	0.06	1322
Biochem.ALP	0.47	0.36	0.03	0.04	1260
Biochem.ALT	0.38	0.00	0.07	0.11	1190
Biochem.AST	0.40	0.00	0.08	0.11	1224
Biochem.Albumin	0.34	0.12	0.07	0.09	1243
Biochem.Calcium	0.64	0.00	0.08	0.11	1253
Biochem.Chloride	0.63	0.00	0.08	0.11	1292
Biochem.Creatinine	0.28	0.00	0.07	0.11	1295
Biochem.HDL	0.49	0.32	0.04	0.05	1186
Biochem.LDL	0.40	0.23	0.05	0.07	1226
Biochem.Phosphorous	0.38	0.06	0.08	0.11	1105
Biochem.Sodium	0.58	0.00	0.08	0.11	1282
Biochem.Tot.Cholesterol	0.32	0.32	0.04	0.04	1259
Biochem.Tot.Protein	0.36	0.00	0.08	0.11	1157
Biochem.Triglycerides	0.43	0.06	0.08	0.11	1092
Biochem.Urea	0.51	0.00	0.08	0.11	1248
BurrowedPelletWeight	0.27	0.31	0.03	0.04	1351
Context.Mean.Freeze	0.42	0.18	0.07	0.08	1024
Cue.Boli	0.21	0.00	0.07	0.11	1072
Cue.Mean.Freeze.Corrected.During	0.40	0.05	0.09	0.12	1008
Cue.Mean.Freeze.Post	0.15	0.19	0.05	0.08	1008
DIRI.DG	0.36	0.17	0.06	0.08	939
EMO	0.27	0.33	0.03	0.04	1342

EPM.ClosedArmDistance	0.38	0.10	0.06	0.09	1298
EPM.ClosedArmTime	0.31	0.27	0.04	0.05	1297
EPM.OpenArmDistance	0.40	0.21	0.05	0.07	1323
EPM.OpenArmEntries	0.37	0.21	0.05	0.07	1323
EPM.OpenArmLatency	0.26	0.29	0.04	0.04	1323
EPM.OpenArmTime	0.39	0.19	0.05	0.07	1323
EarHoleMeanArea	0.47	0.24	0.05	0.06	1297
End.Weight	0.52	0.23	0.05	0.06	1351
FN.Latency	0.28	0.16	0.05	0.07	1324
FN.preWeight	0.63	0.10	0.07	0.09	1358
FPS.Bang.Increase	0.17	0.08	0.06	0.08	1192
FPS.Pre.Bang.Mean	0.46	0.19	0.06	0.08	1192
Glucose.AUC	0.34	0.20	0.06	0.08	984
Glucose.Delta	0.33	0.20	0.06	0.08	985
Glucose_0	0.33	0.00	0.06	0.10	1300
Glucose_15	0.34	0.00	0.07	0.11	1289
Glucose_30	0.40	0.05	0.08	0.11	1284
Glucose_75	0.29	0.21	0.04	0.06	1264
Haem.BASabs	0.27	0.00	0.08	0.11	1167
Haem.HCT	0.25	0.07	0.07	0.10	1166
Haem.HGB	0.28	0.18	0.05	0.08	1164
Haem.LYMabs	0.41	0.13	0.06	0.09	1141
Haem.MCH	0.55	0.32	0.04	0.04	1163
Haem.MCHC	0.65	0.00	0.09	0.11	1159
Haem.MCV	0.50	0.33	0.04	0.04	1174
Haem.MONabs	0.26	0.00	0.07	0.11	1167
Haem.MPV	0.34	0.28	0.05	0.06	1167

Haem.NEUabs	0.42	0.20	0.06	0.08	1167
Haem.PCT	0.25	0.20	0.05	0.07	1137
Haem.PLT	0.31	0.17	0.06	0.08	1158
Haem.RBC	0.31	0.07	0.07	0.11	1163
Haem.RDW	0.41	0.32	0.04	0.05	1156
Haem.WBC	0.39	0.17	0.05	0.08	1167
Imm.CD4CD8Ratio	0.58	0.30	0.05	0.05	1007
Imm.CD4inCD3XGeoMean	0.57	0.18	0.06	0.08	1009
Imm.CD8inCD3YGeoMean	0.66	0.00	0.10	0.13	1006
Imm.PctB220	0.57	0.19	0.06	0.08	1012
Imm.PctCD3	0.45	0.29	0.05	0.06	1010
Imm.PctCD4	0.46	0.20	0.06	0.08	1009
Imm.PctCD4inCD3	0.55	0.30	0.05	0.05	1009
Imm.PctCD8	0.55	0.33	0.04	0.04	1011
Imm.PctNKTAndOutliers	0.44	0.00	0.08	0.13	1003
Insulin.0	0.36	0.00	0.08	0.12	1037
Insulin.15	0.32	0.24	0.05	0.06	1027
Insulin.30	0.32	0.22	0.06	0.07	1021
Insulin.75	0.43	0.01	0.09	0.13	999
Insulin.AUC	0.32	0.18	0.06	0.08	994
Insulin.Delta	0.33	0.20	0.06	0.08	996
OFT.CenterTime	0.31	0.22	0.05	0.06	1346
OFT.Latency	0.27	0.24	0.04	0.05	1346
OFT.TotalActivity	0.32	0.34	0.03	0.04	1344
Obesity.BMI	0.21	0.12	0.05	0.08	1315
Obesity.BodyLength	0.25	0.14	0.05	0.08	1328
PAS.Ambulatory1	0.38	0.13	0.06	0.08	1337

PAS.Ambulatory6	0.33	0.13	0.06	0.08	1327
PAS.TotalAmbulatory	0.45	0.18	0.06	0.07	1336
PAS.TotalFine	0.32	0.23	0.04	0.06	1340
Pleth.EnhancedDiff	0.34	0.30	0.04	0.05	1136
Pleth.Weight	0.59	0.17	0.06	0.08	1314
Pleth.base.BreathFrequency	0.36	0.12	0.06	0.08	1268
Pleth.base.EnhancedPause	0.41	0.00	0.07	0.10	1271
Pleth.base.ExpiratoryTime	0.32	0.11	0.06	0.08	1265
Pleth.base.InspiratoryTime	0.38	0.13	0.06	0.08	1273
Pleth.base.MinuteVolume	0.29	0.16	0.05	0.07	1263
Pleth.base.TidalVolume	0.36	0.10	0.06	0.09	1258
Pleth.meta.BreathFrequency	0.32	0.24	0.05	0.06	1137
Pleth.meta.EnhancedPause	0.35	0.31	0.04	0.05	1145
Pleth.meta.ExpiratoryTime	0.33	0.20	0.05	0.07	1141
Pleth.meta.InspiratoryTime	0.41	0.28	0.04	0.05	1148
Pleth.meta.MinuteVolume	0.29	0.26	0.05	0.06	1139
Pleth.meta.TidalVolume	0.39	0.25	0.05	0.07	1138
Start.Weight	0.65	0.05	0.08	0.10	1378
Weight.GrowthSlope	0.33	0.23	0.04	0.06	1371

Shown are estimates for 97 traits measured in HS mice, attributed to allele sharing via a parent of the same sex h^2_+ and via parents of the opposite sex, h^2_-. $\Delta(h^2_+)$, $\Delta(h^2_-)$ are the estimated standard errors. Heritabilities were estimated by GCTA (Yang, Lee, Goddard, & Visscher, 2011). N is the number of phenotyped mice.

Table S3. Analysis of Variance of Growth in *Man1a2* Mice, Related to Figure 3

**Model**	**Chisq**	**Df**	**Pval**	**Description**
$a + a^2$				quadratic dependence of weight on age
$a + a^2 + s$	163.8	1	10^{-37}	age-independent sex difference
$(a + a^2)*s$	624.2	2	10^{-136}	age-dependent on sex difference
**$(a + a^2)*(s+p)$**	**110.6**	**3**	**10^{-24}**	**parental genotype effect independent of offspring genotype**
$(a + a^2)*(s+p+g)$	5.0	3	0.169	genotype of offspring independent of parental genotype
**$(a + a^2)*(s+p*g)$**	**17.2**	**3**	**0.00062**	**interaction between parental and offspring genotype (POE)**
$(a + a^2)*(s*p +p*g)$	14.3	3	0.00243	sex-specific parental effect
$(a + a^2)*(s*p*g)$	3.4136	6	0.75542	sex-specific parental and POE effects

A series of nested linear mixed models of increasing complexity were fitted to weights of 108 offspring from a reciprocal cross of the Man1a2 knockout. A total of 855 weight measurements were taken at ages between 4 and 11 weeks. Each line describes the fit of a model that also included a random effect for each mouse in addition to the fixed effects described in the Model column. Here a: age in weeks, s: sex, p: parental genotype (either maternal KO or maternal WT), g: offspring genotype (KO or WT). Models with an * contain interaction terms. Each model is nested inside all the models beneath it. Chisq is the chi-squared statistic for the additional fit of the model compared to the model on the line immediately above. Df is the degrees of freedom (number of additional parameters) in the test of the model, and Pval is the p-value of the test of the null hypothesis that the model explains no more variation than the nested model above it. Description is a verbal explanation of the model. The Model "interaction between parental and offspring genotype (POE)" highlighted in bold corresponds to the curves in Figure 3.

Table S5. Analysis of Variance for *H2-ab1* Reciprocal Crosses, Related to Figure 4

**(a) $CD4^+$ homozygous**	**Df**	**Sum Sq**	**P-value**
s	1	140.73	**0.0009**
p	1	97.32	**0.005**
s*p	1	16.72	0.21
Residuals	34	360.2	
Total	37	614.97	

**(b) $CD4^+$ heterozygous**	**Df**	**Sum Sq**	**P-value**
s	1	69.94	**0.006**
g	1	690.67	**7E-13**
p	1	2.66	0.58
s*g	1	1.61	0.66
s*p	1	10.19	0.28
g*p	1	49.45	**0.020**
s*g*p	1	11.78	0.25
Residuals	65	565.39	
Total	72	1401.69	

**(c) DCX heterozygous**	**Df**	**Sum Sq**	**P-value**
s	1	997	0.35
g	1	2698	0.13
p	1	603	0.46
s*g	1	103	0.76
s*p	1	2963	0.11
g*p	1	1313	0.28
s*g*p	1	6818	**0.020**
Residuals	22	24167	
Total	29	39662	

(a) $CD4^+$ cells (blood), homozygous cross. (b) $CD4^+$ cells (spleen), heterozygous cross (c) Adult neurogenesis (DCX), heterozygous cross. s , g are sex and *H2-ab1* genotype of F1 mice; p is the parental *H2-ab1* genotype (maternal vs paternal KO). Shown are the variation (Sum Sq) of each effect and their interactions; p: parental effect (difference between a mother or father carrying the *H2-ab1* KO), g: effect of the *H2-ab1* KO in the F1, g*p: parent of origin effect, s*g*p: sex-dependent parent of origin effect. In (a) all F1 mice are heterozygous. P-values are from F-tests; those <0.05 are in bold.

Table S7. Known Imprinted Genes Differentially Expressed in the *H2-ab1* Reciprocal Cross, Related to Figure 4E

**tissue**	**sex**	**type**	**gene**	**pat**	**mat**	**Fold Change**	**FDR**
L	F	Het	Bcl2l1	1300	746	0.57	4.49E-03
L	M	Het	H19	41	9	0.22	3.44E-03
L	F	Wt	Mkrn1-ps1*	8	35	4.44	4.98E-07
L	F	Wt	Bcl2l1	1081	669	0.62	5.15E-03
L	M	Wt	H19	2	42	15.75	1.56E-08
L	M	Wt	Dcn	489	796	1.63	4.06E-02
H	F	Het	Igf2	736	263	0.36	2.93E-18
H	F	Het	Dlk1	71	30	0.42	5.14E-04
H	F	Het	Cdkn1c	64	32	0.51	2.71E-02
H	M	Het	Xlr3b	1	15	11.23	7.77E-03
H	M	Het	Meg3	5734	9441	1.65	8.44E-03
H	F	Wt	Dlk1	58	26	0.45	8.11E-03
H	M	Wt	Dcn	264	119	0.45	7.35E-05
H	M	Wt	Dio3	87	31	0.37	1.68E-03
H	M	Wt	Meg3	8489	12307	1.45	3.99E-02
H	M	Wt	Plagl1	180	72	0.40	6.28E-03
H	M	Wt	Mirg	71	141	1.98	4.90E-03

Genes are categorized by tissue (L, lung; H, hippocampus), sex (M, male; F, female) and type (whether the F1 mouse was wild-type or heterozygous for the KO). Shown are the mean expression levels in animals with paternal (pat) or maternal (mat) KO (based on three or four biological replicates), the fold-change in expression, log2 fold-change and FDR of the difference, as computed by DESeq (Anders & Huber, 2010). Note that the imprinted status of Mkrn1-ps1* is disputed(Gray, Wilson, Fortin, & Nicholls, 2006; Hirotsune et al., 2003).

Table S8. Counts of Differentially Expressed Genes in Common between Experimental Conditions in the Heterozygous *H2-ab1* Reciprocal Cross, for (a) Lung and (b) Hippocampus, Related to Figure 5E

(a) lung

	Het.F	Het.M	WT.F	WT.M
**Het.F**	164	1	32	33
**Het.M**	-2	54	10	13
**WT.F**	-50	-8	1210	219
**WT.M**	-49	-15	-200	616

(b) hippocampus

	Het.F	Het.M	WT.F	WT.M
**Het.F**	124	4	6	7
**Het.M**	-3	26	3	3
**WT.F**	-3	0	74	11
**WT.M**	-1	0	-5	97

The negative counts in the lower triangle of each table are for genes in common but differentially expressed in opposite directions (the direction of expression is the sign of then difference between mice whose father was KO and mice whose mother was KO). The positive counts in the upper triangles are numbers in common and differentially expressed in the same direction.
